# Adult-onset inflammatory linear verrucous epidermal nevus successfully treated with intralesional steroid

**DOI:** 10.1016/j.jdcr.2024.02.001

**Published:** 2024-02-10

**Authors:** Austin N. Johnson, Katie Sum, Kerri E. Rieger, Albert S. Chiou, Dayan J. Li

**Affiliations:** Department of Dermatology, Stanford University School of Medicine, Stanford, California

**Keywords:** ILK, ILVEN, inflammatory linear verrucous epidermal nevus, intralesional steroid

## Introduction

Inflammatory linear verrucous epidermal nevus (ILVEN) is a rare dermatosis presenting as pruritic, hyperkeratotic, and erythematous papules and plaques arranged along the lines of Blaschko. Histologically, it is characterized by epidermal acanthosis with alternating para- and orthokeratosis and a perivascular lymphocytic infiltrate.^1^ It usually appears in children,^2^ with only 19 adult cases reported in literature.^1^ ILVEN harbors features of both a hamartoma and an inflammatory disorder. Initial descriptions of its early onset, histology, and recalcitrance to therapy helped classify it as a type of epidermal nevus.^2^ Recently, identification of lesional postzygotic *HRAS* and *KRT10* mutations associated with elevated interleukin (IL)-1, IL-6, and tumor necrosis factor-alfa or germline *CARD14* mutation nominates ILVEN as a heterogeneous group of mosaic genodermatoses arising in an autoinflammatory background.^1^^,^^3^ The lesions are often recalcitrant to treatment, with anecdotal reports of improvement with lasers, topical antiinflammatory agents, and surgical excision.^4^ Here, we describe a patient with adult-onset ILVEN successfully treated with intralesional triamcinolone acetonide (ILK).

## Case report

A 60-year-old woman presented with a 1-year history of a persistent pruritic rash on the upper portion of her right trunk and right side of her upper extremity 1 month after shingles vaccination on the right arm. Besides a remote history of a scaly rash on the left shin that resolved with topical steroids, she had no other mucocutaneous lesions, prior exposure to topical agents, medication changes, or B symptoms. Previous treatments with topical antiinflammatory agents including calcipotriene, tacrolimus, tapinarof, and betamethasone ointment under occlusion were unsuccessful.

**Fig 1:**
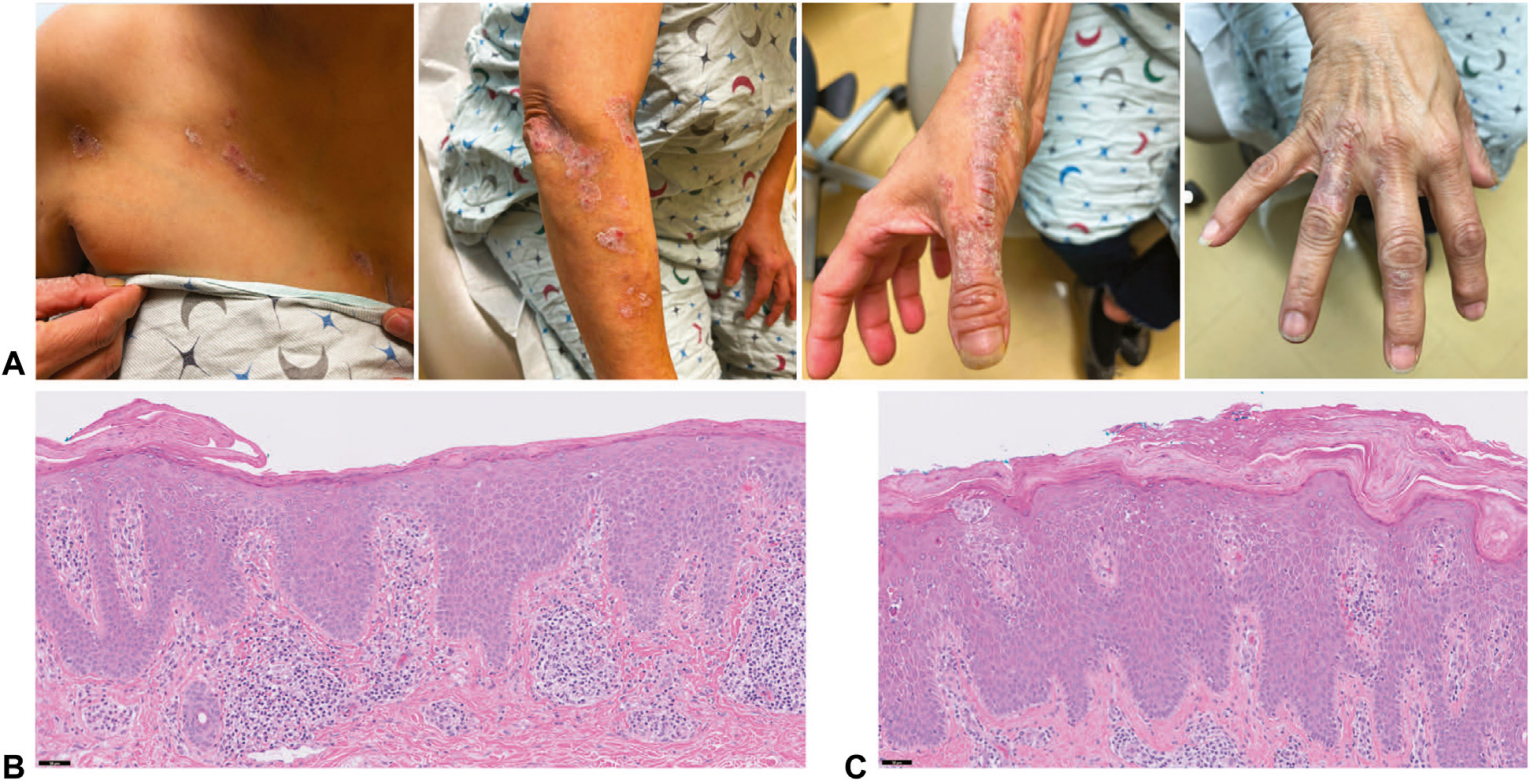
Initial inflammatory linear verrucous epidermal nevus presentation. **A**, Linearly arranged erythematous to violaceous scaly papules and plaques in a Blaschkoid distribution spanning the right chest and the right side of the upper extremity. Hematoxylin and eosin staining of lesions biopsied from the right chest (**B**) and right wrist (**C**) showing epidermal acanthosis with horizontally alternating ortho- and parakeratosis, marked epidermal spongiosis, and perivascular lymphocytic infiltrate (scale bar, 50 μm).

Examination revealed linearly arranged erythematous to violaceous scaly papules and plaques extending from the right chest all the way to the distal dorsal aspect of the fingers in a Blaschkoid distribution (Fig 1, *A*). Punch biopsies of the right chest and right wrist lesions showed epidermal acanthosis with prominent spongiosis, serum crust, horizontally alternating para- and orthokeratosis, and perivascular lymphocytic infiltrate in the papillary dermis without a lichenoid infiltrate or eosinophils (Fig 1, *B*, *C*). Her clinicopathologic presentation supported a diagnosis of ILVEN over blaschkitis, linear psoriasis, linear lichen planus, and lichen striatus.

**Fig 2:**
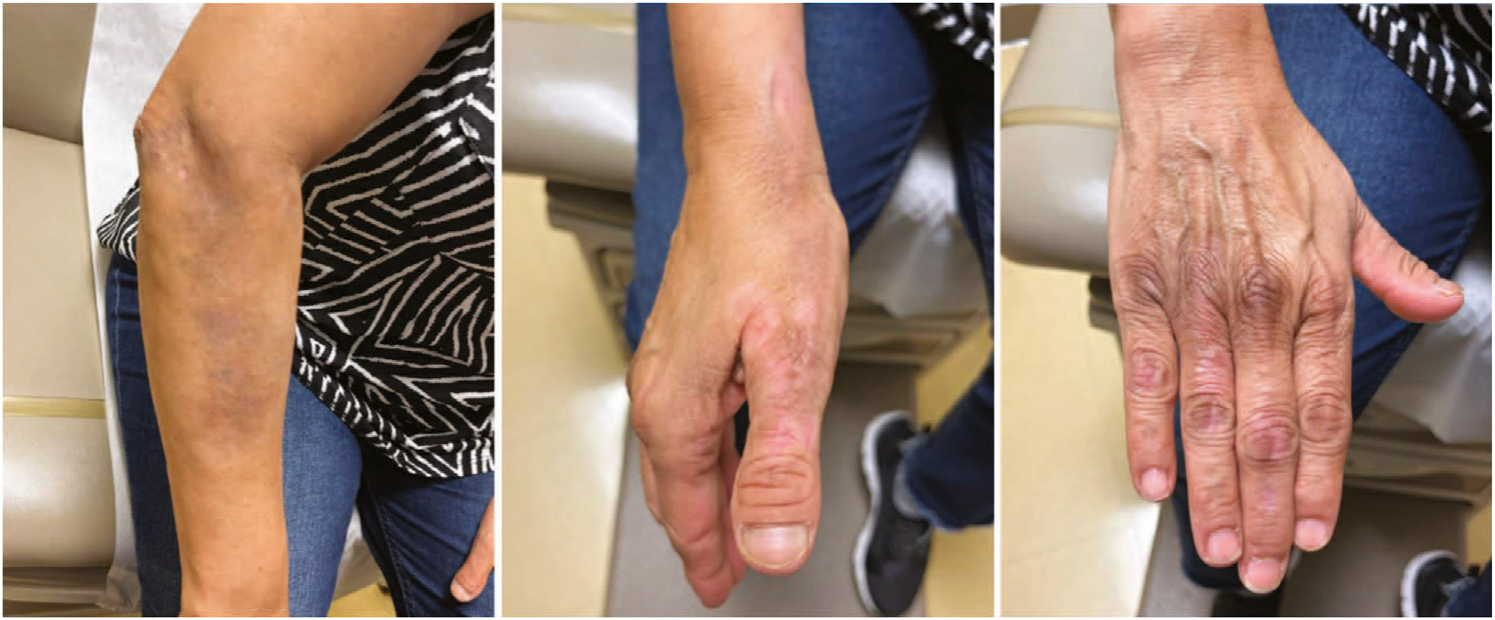
Inflammatory linear verrucous epidermal nevus (ILVEN) improvement after intralesional triamcinolone acetonide (ILK). Marked reduction of erythema and scale of ILVEN lesions on the right forearm and dorsal aspect of the right hand 2 to 6 months after single injections of 10 to 20 mg/mL of ILK.

Given the patient’s lack of response to repeat months-long topical steroid and excimer laser, 20 mg/mL of ILK (1 mL total) was administered to lesions on the right thumb and lateral aspect of the dorsal right hand. Topical 2% tofacitinib cream applied twice daily was also started about a month following ILK, but was discontinued after 1 month because of its cost and minimal antipruritic effects. Flattening and improvement of erythema of treated areas occurred within 1 to 2 days after single steroid injections, progressing to complete resolution without recurrence on 2-month follow-up (Fig 2). Subsequent ILK (1-2 mL of 20 mg/mL) injections into the remaining right hand and right side of the upper extremity plaques over several clinic visits led to similar rapid and lasting improvement without cutaneous atrophy (Fig 2). Eight months after the 1-time ILK administration, a mild recurrence was noted over her lateral aspect of the dorsal right hand, which promptly responded to a second injection (2-3 mL of 10 mg/mL ILK). The patient has continued to exhibit durable response to ILK as the sole means of therapy, supplemented only by emollients applied daily over the treated areas. Follow-up visits every 6 months are planned to assess her need for repeat injections.

## Discussion

Examples of blaschkolinear dermatoses presenting in adulthood include ILVEN, blaschkitis, and linear psoriasis. Although it is often difficult to distinguish among these conditions, adult-onset ILVEN presents with certain distinct clinical and histologic features. The characteristic scaly lesions of ILVEN persist usually for >1 year, unlike the papules and vesicles observed in blaschkitis that commonly self-resolve within 2 months of onset.^5^^,^^6^ Histologically, ILVEN features alternating para- and orthokeratosis and a perivascular lymphocytic infiltrate, which contrasts to the confluent parakeratosis with intracorneal and subcorneal neutrophils that characterize psoriasis and the lichenoid infiltrate with dyskeratotic keratinocytes and pigment incontinence seen in blaschkitis.^1^^,^^7^ Also unlike blaschkitis and psoriasis, ILVEN is often recalcitrant to therapy, with only sparse reports of improvement with various topical agents, lasers, and surgical excision.^1^^,^^4^ All of these aforementioned clinicopathologic features supported a diagnosis of ILVEN for our patient.

To our knowledge, successful and durable treatment of ILVEN with ILK has rarely been reported, with the few previous cases requiring up to biweekly injections followed by recurrence within months after therapeutic discontinuation.^8^ In this case, the close temporal association between ILK and improvement of the lesions at the sites of injection supports its therapeutic role for this difficult-to-treat condition. Notably, sustained improvement of the lesions occurred after just 1 injection despite discontinuation of prior therapies. We postulate that intralesional steroid, in addition to directly inhibiting epidermal hyperplasia, effectively interrupts the local autoinflammatory cycle by dampening the production of cytokines (eg, IL-1, IL-6, and tumor necrosis factor-alfa) that promote keratinocyte hyperproliferation in genetically predisposed individuals (eg, those with underlying mosaic keratinocyte mutations).^9^ In the patient’s case, the preceding zoster vaccine may have been a trigger for local immune activation. With superior penetration and dermal concentration than topical modalities, intralesional approaches (using antiinflammatory or antimitotic agents) may be efficacious strategies in mitigating the loco-regional persistence of such lesions. In light of its rapid and durable therapeutic efficacy and minimal adverse effects, ILK can be considered a first-line option for treating ILVEN in adults.

## Conflicts of interest

None disclosed.
